# Supplementary material for: Three corrections for overshoot effect improved the dose for step-and-shoot intensity-modulated radiation therapy
Source: PLoS One. 2021 Apr 23;16(4):e0250243. doi: 10.1371/journal.pone.0250243 (PMC8064569; doi:10.1371/journal.pone.0250243)
Supplement: Attachment — Submitted filename: Response to Reviewers.docx [file pone.0250243.s007.docx]

Journal Requirements:

When submitting your revision, we need you to address these additional

requirements.

1. Please ensure that your manuscript meets PLOS ONE's style

requirements, including those for file naming.

ANS: Yes, we did.

2. We suggest you thoroughly copyedit your manuscript for language

usage, spelling, and grammar. If you do not know anyone who can help you

do this, you may wish to consider employing a professional scientific

editing service.

ANS: Yes, we did. As the attached file, ***

Reviewer's Responses to Questions

Comments to the Author

1. Is the manuscript technically sound, and do the data support the

conclusions?

Reviewer #1: Yes

ANS: Thank Reviewer.

2. Has the statistical analysis been performed appropriately and

rigorously?

Reviewer #1: Yes

ANS: Thank Reviewer.

3. Have the authors made all data underlying the findings in their

manuscript fully available?

Reviewer #1: Yes

ANS: Thank Reviewer.

4. Is the manuscript presented in an intelligible fashion and written in

standard English?

Reviewer #1: Yes

ANS: Thank Reviewer.

5. Review Comments to the Author

Reviewer #1: This is an interesting topic. The authors measured and

listed the corrections for overshoot effect in step-and-shoot IMRT

delivery.

ANS: Thank Reviewer.

Overshoot effect (multiple segments per field) cannot be caught from end

effect test (one segment per field) in machine QA. And clinical

physicists should know the limits of the machines when applying

optimization parameters in TPS.

ANS: We agree.

Clinically, there are two ways to avoid or reduce overshoot effect in

most TPS by

1. increasing minimum segment MU and

2. limiting total number of segments per beam (increase minimum segment

MU) during plan optimization.

Probably it can be mentioned in discussion.

ANS: Thank Reviewer a lot. We did in new manuscript.

For clinical plans which have multiple fields, very few segments, and

low MUs, overshoot effect might be significant and lower GAMMA passing

rate might be found.

ANS: We agree.

Typo: line 135: should be "MU/min", not "MJ/min"

ANS: Thank Reviewer a lot. I changed "MJ/min" to "MU/min" at line 135 in new manuscript.

6. PLOS authors have the option to publish the peer review history of their article (what does this mean? [3]). If published, this will include your full peer review and any attached files.

If you choose "no", your identity will remain anonymous but your review may still be made public.

Do you want your identity to be public for this peer review? For information about this choice, including consent withdrawal, please see our Privacy Policy [4].

Reviewer #1: No

ANS: We respect to Reviewer’s option.
