# Supplementary material for: Three corrections for overshoot effect improved the dose for step-and-shoot intensity-modulated radiation therapy
Source: PLoS One. 2021 Apr 23;16(4):e0250243. doi: 10.1371/journal.pone.0250243 (PMC8064569; doi:10.1371/journal.pone.0250243)
Supplement: Attachment — Submitted filename: Response to Reviewers.docx [file pone.0250243.s008.docx]

Reviewers' comments:

Reviewer's Responses to Questions

Comments to the Author

1. If the authors have adequately addressed your comments raised in a previous round of review and you feel that this manuscript is now acceptable for publication, you may indicate that here to bypass the "Comments to the Author" section, enter your conflict of interest statement in the "Confidential to Editor" section, and submit your "Accept" recommendation.

Reviewer #1: All comments have been addressed

ANS: Thank Reviewer.

-------------------------

2. Is the manuscript technically sound, and do the data support the conclusions?

The manuscript must describe a technically sound piece of scientific research with data that supports the conclusions. Experiments must have been conducted rigorously, with appropriate controls, replication, and sample sizes. The conclusions must be drawn appropriately based on the data presented.

Reviewer #1: Yes

ANS: Thank Reviewer.

-------------------------

3. Has the statistical analysis been performed appropriately and rigorously?

Reviewer #1: Yes

ANS: Thank Reviewer.

-------------------------

4. Have the authors made all data underlying the findings in their manuscript fully available?

The PLOS Data policy [1] requires authors to make all data underlying the findings described in their manuscript fully available without restriction, with rare exception (please refer to the Data Availability Statement in the manuscript PDF file). The data should be provided as part of the manuscript or its supporting information, or deposited to a public repository. For example, in addition to summary statistics, the data points behind means, medians and variance measures should be available. If there are restrictions on publicly sharing data--e.g. participant privacy or use of data from a third party--those must be specified.

Reviewer #1: Yes

ANS: Thank Reviewer.

-------------------------

5. Is the manuscript presented in an intelligible fashion and written in standard English?

PLOS ONE does not copyedit accepted manuscripts, so the language in submitted articles must be clear, correct, and unambiguous. Any typographical or grammatical errors should be corrected at revision, so please note any specific errors here.

Reviewer #1: Yes

ANS: Thank Reviewer.

-------------------------

6. Review Comments to the Author

Please use the space provided to explain your answers to the questions above. You may also include additional comments for the author, including concerns about dual publication, research ethics, or publication ethics. (Please upload your review as an attachment if it exceeds 20,000 characters)

Reviewer #1: From clinical plan perspective, not every step-and shoot IMRT plan should be corrected. Can author address more on the examples/plans that impacted by overshoot effect. For example, a publication, The step‐and‐shoot IMRT overshooting phenomenon: a novel method to mitigate patient overdosage. by Heming Zhen etc., found the correction can improve prostate plan QA passing rate. And another publication, Clinical implications of the overshoot effect for treatment plan delivery and patient-specific quality assurance for step-and-shoot IMRT. by John A. Baines etc., compared prostate and head and neck plans. QA passing rate can be improved with these corrections. But is it necessary to apply the correction for all step-and-shoot plan? Or if other methods used during plan optimization, like increasing minimum segment MU or reducing dose rate, can the correction can be waived?

ANS: Honestly, I have to confess that the plans with overshoot effect are much fewer, even served all cases in last year in our department. I also agree that overshoot effect was minimized as increasing segment MU or reducing dose rate. I also believe that increasing segment MU or reducing dose rate is most efficient to minimize overshoot effect. The above opinions were reported in our manuscript.

Block correction exclude overshoot and undershoot segments. Reverse-sequence correction, and index correction are compensate overshoot or undershoot segments to each other. Theoretically, overshoot effect could be reduce to zero with the three corrections, not only minimize overshoot effect. Unfortunately, before the corrections will be applied, corrections have to be approved by government. The other problem is time consuming for calculating the index of each segment and re-arranging the order of all segment. A computer program have to design for the problem.

I have to emphasize that the purpose in this report is evaluated and analyzed the availability of the three correction before the corrections are applied for clinical cases. I also have to emphasize that increasing segment MU or reducing dose rate is most efficient to minimize overshoot effect. I also believe that the result of overshoot effect as increasing segment MU or reducing dose rate is acceptable in clinical.

-------------------------

7. PLOS authors have the option to publish the peer review history of their article (what does this mean? [2]). If published, this will include your full peer review and any attached files.

If you choose "no", your identity will remain anonymous but your review may still be made public.

Do you want your identity to be public for this peer review? For information about this choice, including consent withdrawal, please see our Privacy Policy [3].

Reviewer #1: No

ANS: We respect to Reviewer’s option.
